# Supplementary figures and images for: Increased urea nitrogen salvaging by a remodeled gut microbiota helps nonhibernating pikas maintain protein homeostasis during winter
Source: PLoS Biol. 2025 Oct 16;23(10):e3003436. doi: 10.1371/journal.pbio.3003436 (PMC12530534; doi:10.1371/journal.pbio.3003436)

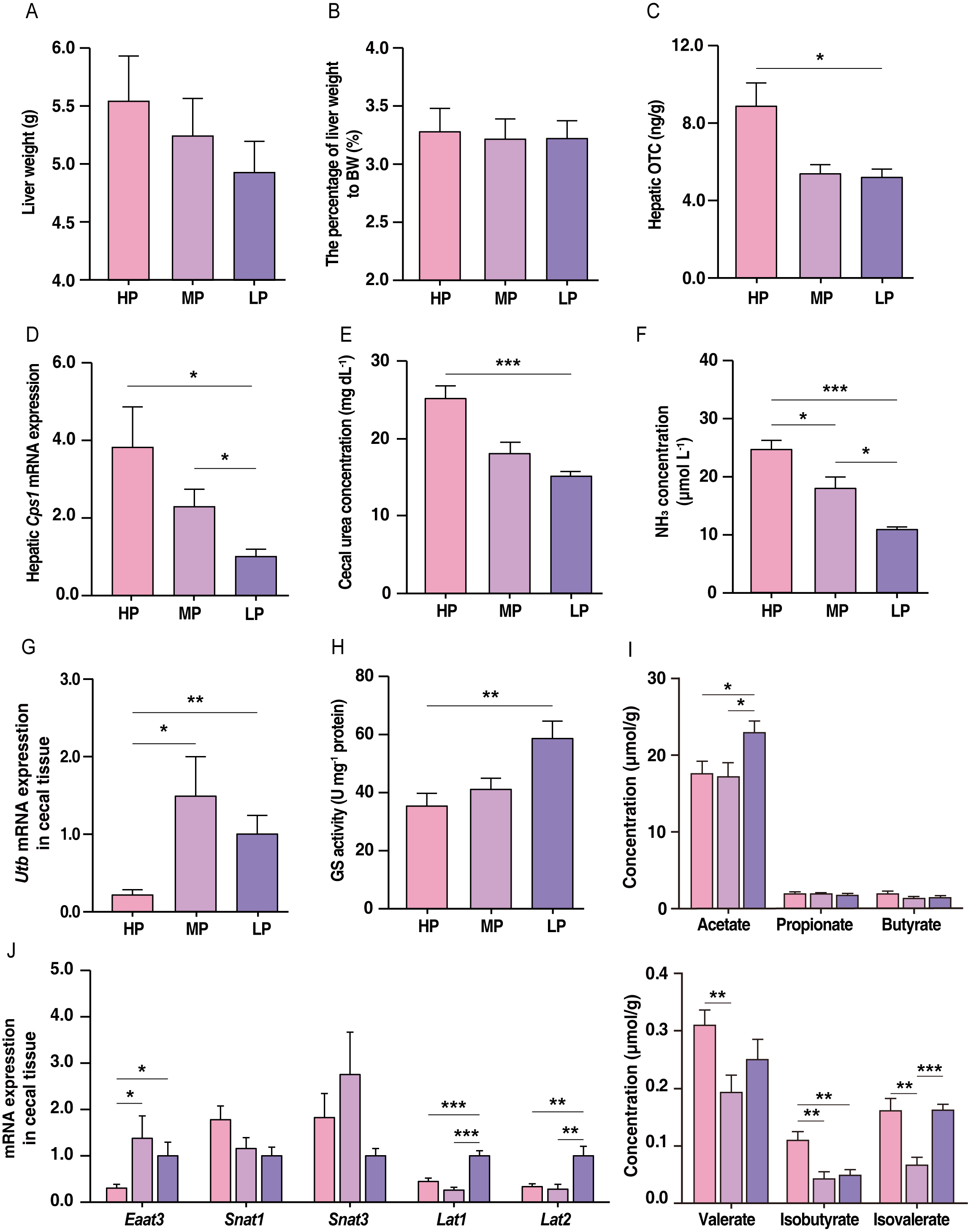

Supplement: S1 Fig — (A) Liver weight from pikas fed high-protein (HP), medium-protein (MP), or LP diets (n = 8 per group). (B) The ratio of liver weight to body weight (BW). (C) Relative mRNA expression of Cps1 in the liver, as determined by qRT-PCR. (D) Protein abundance of ornithine transcarbamylase (OTC) in the liver, as determined by Elisa. (E) Urea concentration in cecal contents. (F) NH3 concentration in cecal contents. (G) Liver glutamine synthetase (GS) activity in liver. (H) Relative mRNA expression of the urea transporter Utb expression in cecal epithelium. (I) Concentration of short chain fatty acids (SCFAs), including acetate, propionate, butyrate, iso-butytate, iso-valerate and valerate in cecal contents. (J) Relative mRNA expression of key amino-acid transporter genes in cecal epithelium. All data are presented as mean ± SEM (n = 8 pikas per group). Statistical significance was determined by ANOVA with post-hoc Tukey’s test. Asterisks denote significant differences among the three groups (*p < 0.05; **p < 0.01; ***p < 0.001). The numerical data used to generate the graphs in this figure are available in S8 Data. (TIF) [file pbio.3003436.s001.tif]

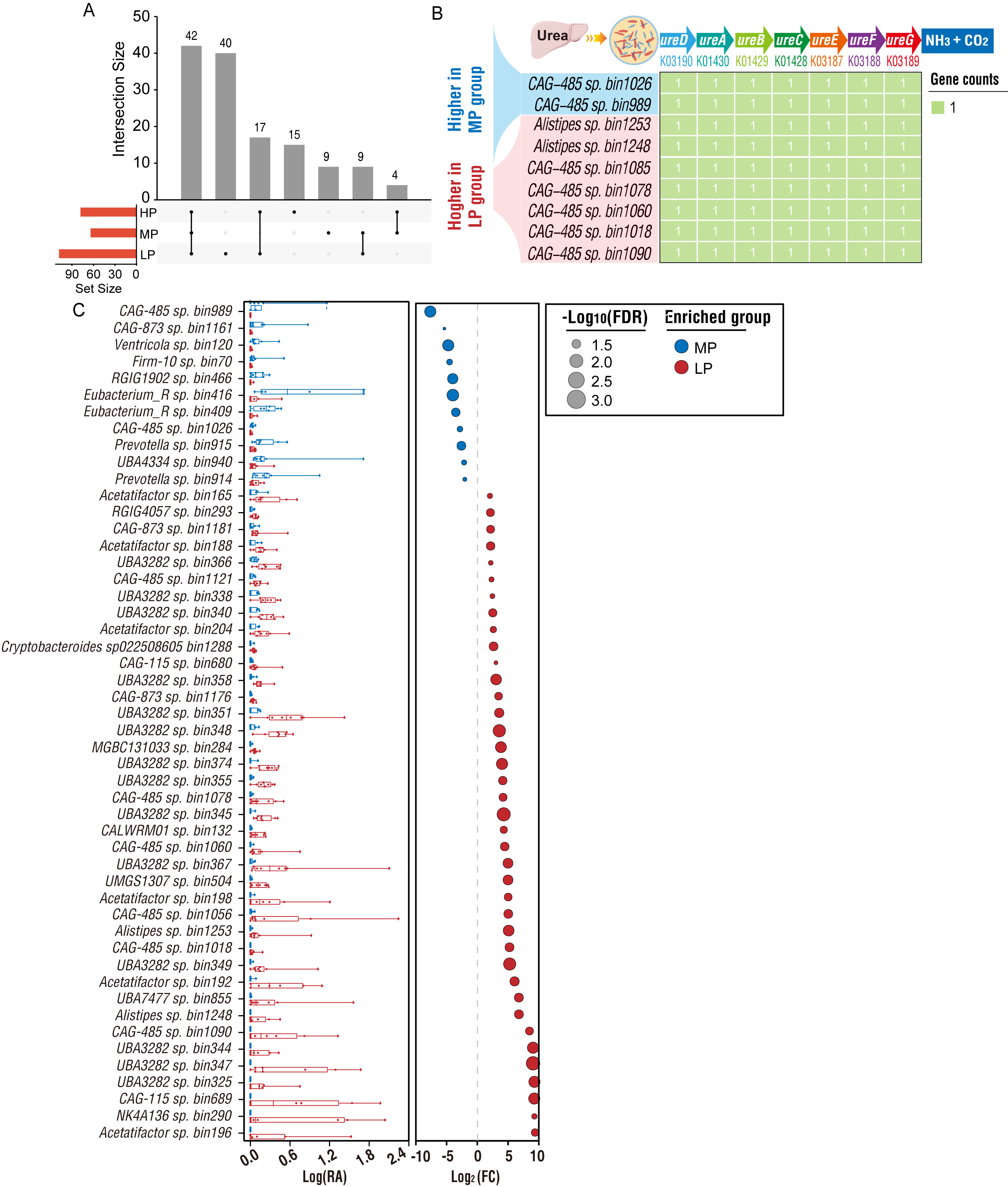

Supplement: S2 Fig — (A) Upset plot visualizing the intersections of urease-encoding MAGs among the HP, MP, and LP groups. The bar charts represent the size of each intersection (top) and the total number of MAGs per group (left). (B) Comparison of the differential abundance of MAGs from MP pikas versus LP pikas. The left border represents log-transformed relative abundance (RA, %) of MAGs. The right border represents log-transformed fold-change values of RA. The RA of MAGs between two groups was tested with the nonparametric Wilcoxon test with an FDR-corrected p-value (MAGs with FDR < 0.05 are shown). (C) The potential of group-enriched MAGs for identifying enzymes encoded by urease homologs (ureA, ureB, ureC, ureD, ureE, ureF, and ureG) in MP and LP pikas. The numerical data used to generate the graphs in this figure are available in S9 Data. (TIF) [file pbio.3003436.s002.tif]

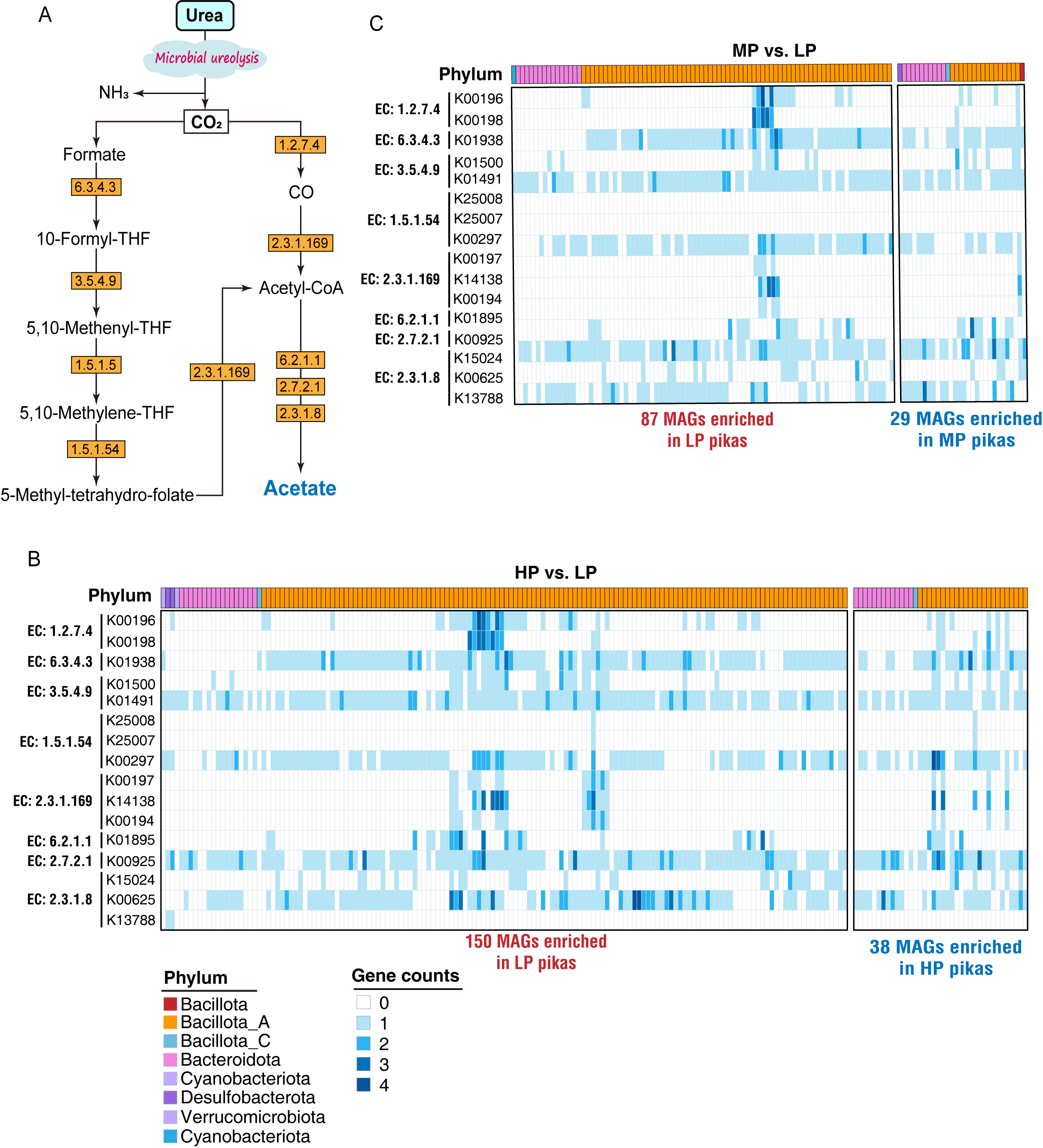

Supplement: S3 Fig — (A) Schematic overview of major CO2 fixation pathways and their key microbial enzymes. (B) Different MAGs that encode enzymes for acetogenesis in CO2 fixation pathways between HP and LP pikas. (C) Different MAGs that encode enzymes of acetogenesis in CO2 fixation pathways between MP and LP pikas. Differences in the relative abundance (RA) of MAGs between two groups were tested with the nonparametric Wilcoxon test with an FDR-corrected p-value (MAGs with FDR < 0.05 are shown). The numerical data used to generate the graphs in this figure are available in S10 Data. (TIF) [file pbio.3003436.s003.tif]

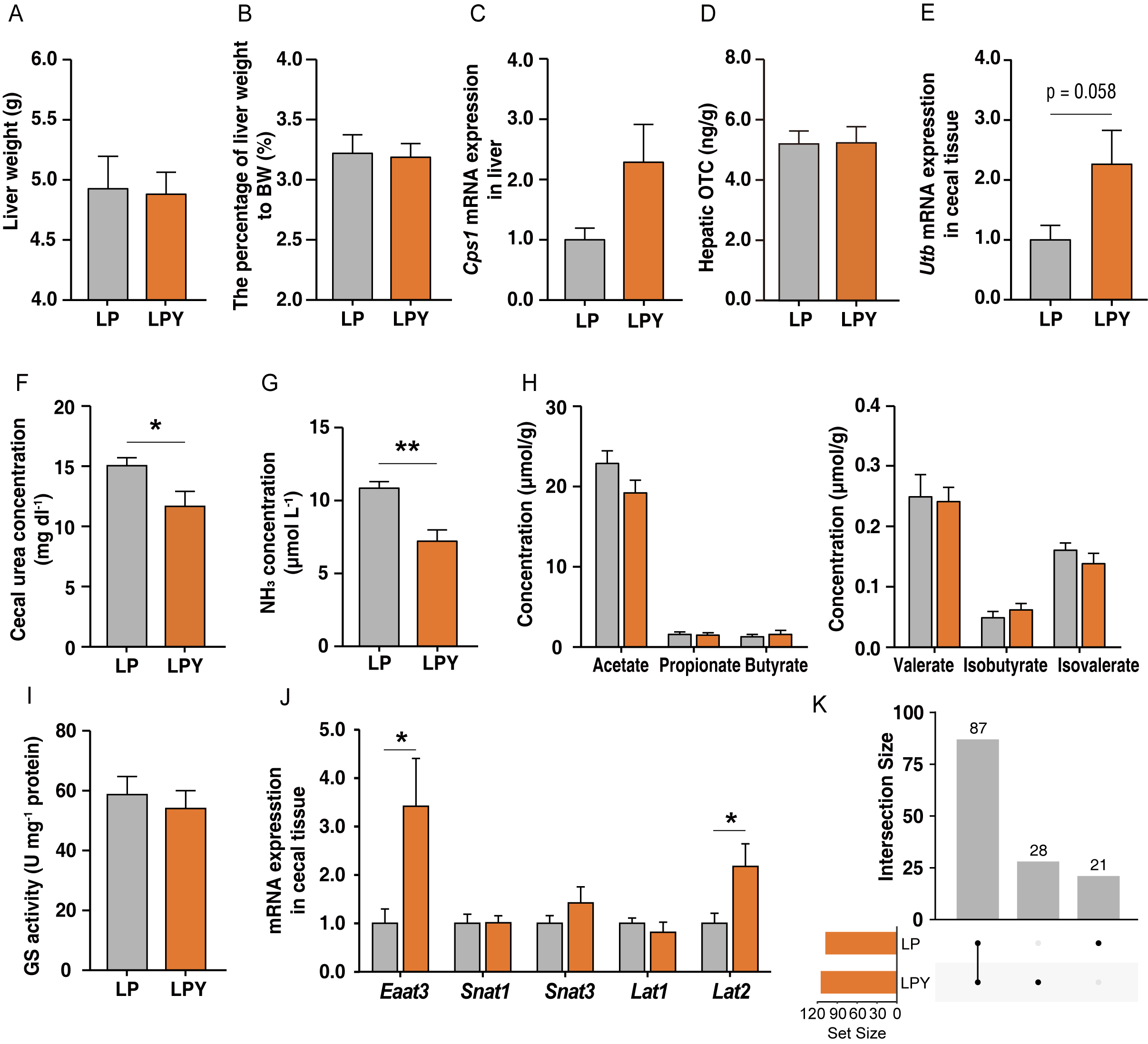

Supplement: S4 Fig — (A) Liver weight from pikas in the low-protein (LP) and supplementation of the diet with yak fecal bacteria (LPY diet). (B) The ratio of liver weight to body weight (BW). (C) Relative mRNA expression of Cps1 in the liver, as determined by qRT-PCR. (D) Protein abundance of ornithine transcarbamylase (OTC) in the liver, as determined by Elisa. (E) Urea concentration in cecal contents. (F) NH3 concentration in cecal contents. (G) Liver glutamine synthetase (GS) activity in liver. (H) Relative mRNA expression of the urea transporter Utb expression in cecal epithelium. (I) Concentration of short chain fatty acids (SCFAs), including acetate, propionate, butyrate, iso-butytate, iso-valerate and valerate in cecal contents. (J) Relative mRNA expression of key amino-acid transporter genes in cecal epithelium (K) Upset plot visualizing the intersections of urease-encoding MAGs between the LP and LPY groups. The bar charts represent the size of each intersection (top) and the total number of MAGs per group (left). All data represent the mean ± SEM (n = 8 pikas per group). Statistical significance was determined by the two-tailed Student t test. Asterisks denote significant differences between the two groups (*p < 0.05; **p < 0.01; ***p < 0.001). The numerical data used to generate the graphs in this figure are available in S11 Data. (TIF) [file pbio.3003436.s004.tif]

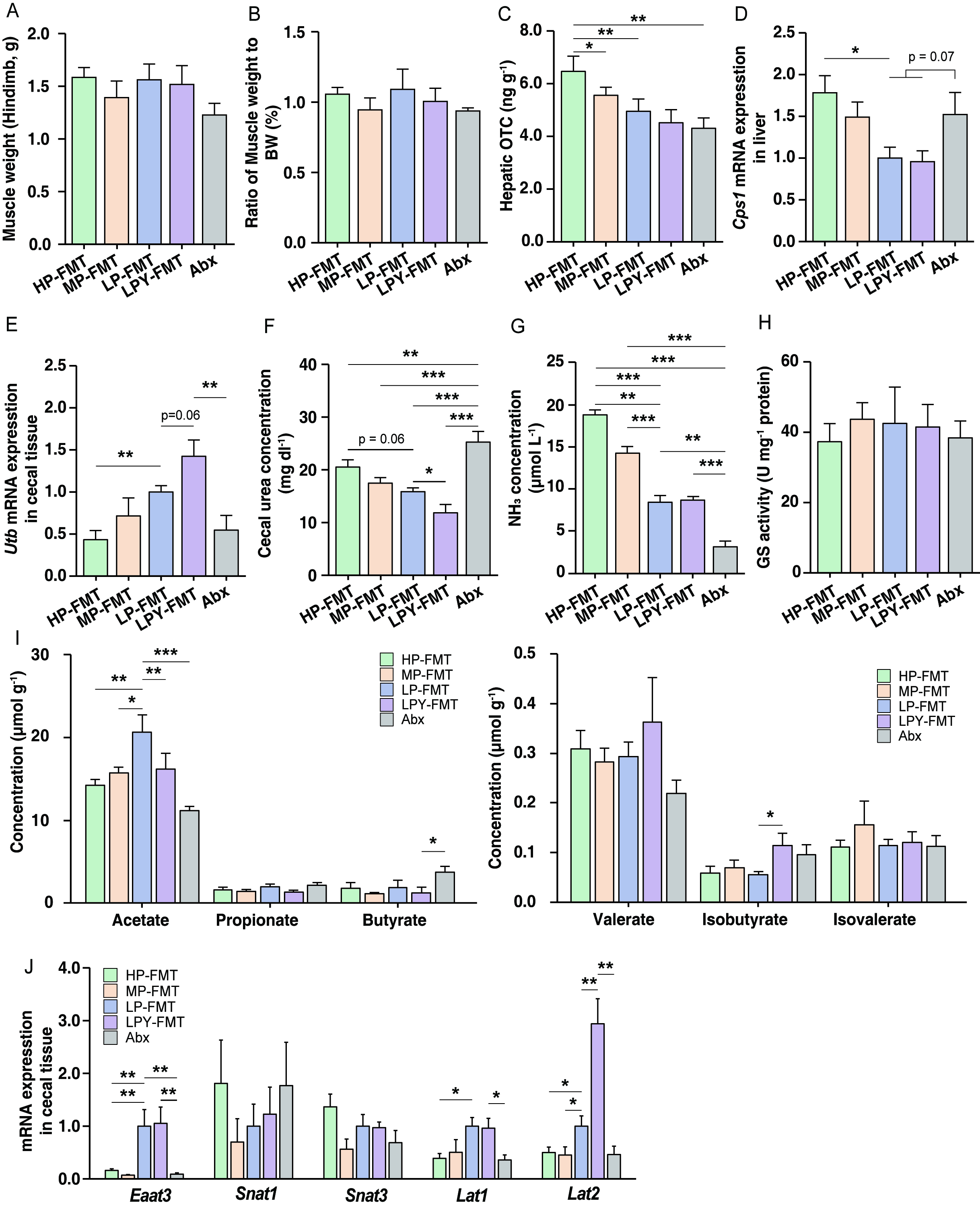

Supplement: S5 Fig — (A) Muscle weight. (B) Ratio of muscle weight to BW. (C) Effects of hepatic urea synthesis on the concentration of OTC in liver. (D) Hepatic urea synthesis of Cps1 expression. (E) Utb expression in cecal epithelium. (F) Cecal urea concentration. (G) NH3 concentration in cecal. (H) Liver glutamine synthetase (GS) activity in liver. (I) Concentration of short-chain fatty acids (SCFAs) in cecal. (J) Expression of genes encoding amino-acid transporters in cecal epithelium. Two experiments are included in this part; one compared metabolic phenotypes and microbial differences induced by different protein diets via FMT experiments (HP-FMT, MP-FMT, LP-FMT, and Abx pikas), and the other assessed the effect of supplementation with yak fecal bacteria on metabolic phenotypes and microbes induced by the LP (LP-FMT, LPY-FMT, and Abx pikas). The data for LP-FMT and Abx were shared in these two experiments. All data represent the mean ± SEM. N = 6 pikas except Abx pikas (n = 5). Statistical significance was determined by one-way ANOVA with Tukey’s post-hoc test, where asterisks indicate a significant difference (p < 0.05) for the comparison among HP-FMT, MP-FMT, LP-FMT, and Abx groups; for the comparison among LP-FMT, LPY-FMT, and Abx groups, asterisks denote significance at three levels (*p < 0.05, **p < 0.01, ***p < 0.001). The numerical data used to generate the graphs in this figure are available in S12 Data. (TIF) [file pbio.3003436.s005.tif]

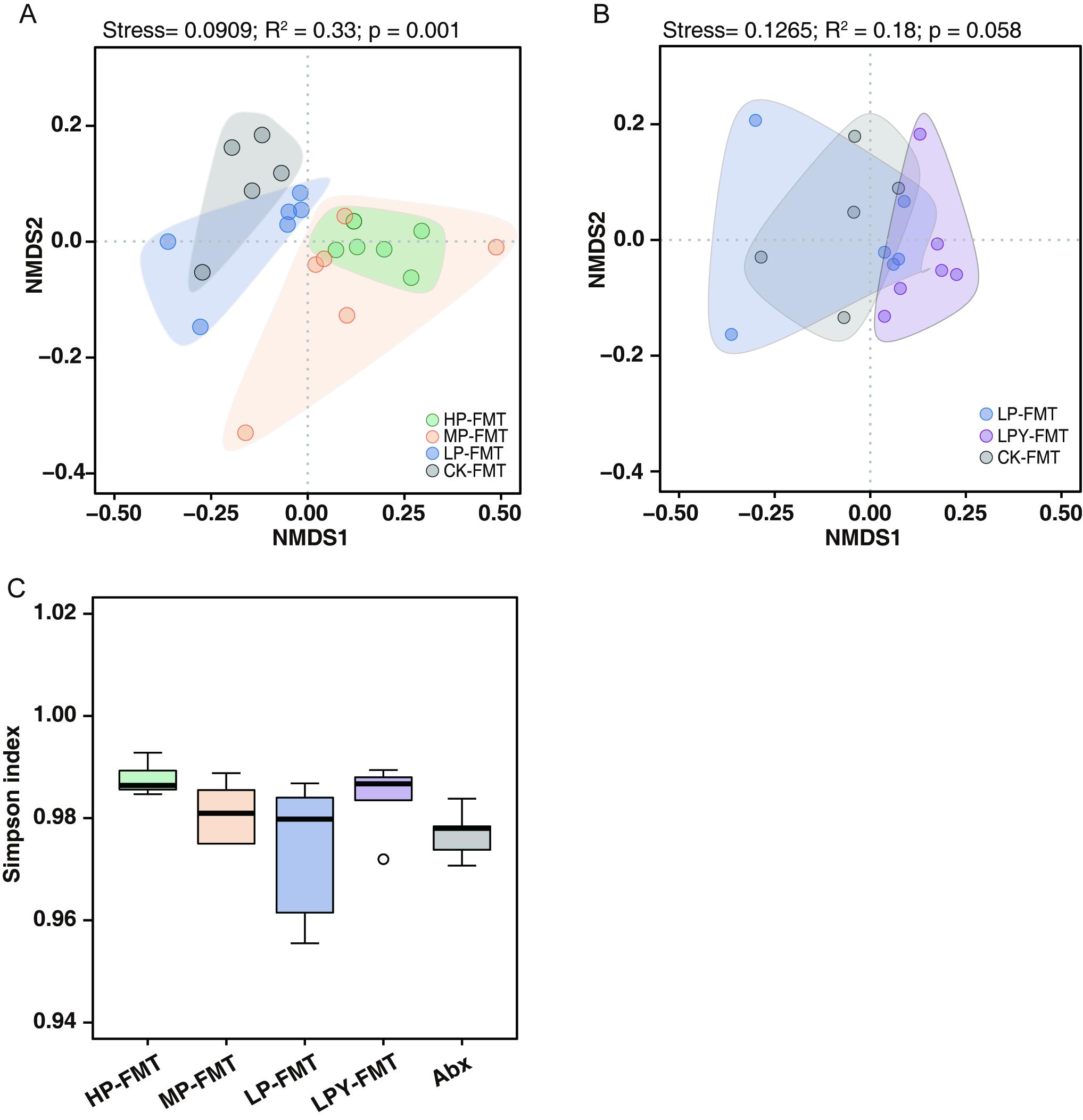

Supplement: S6 Fig — (A) Nonmetric multidimensional scaling (NMDS) plot of Bray–Curtis dissimilarities, visualizing community structure differences among HP-FMT, MP-FMT, LP-FMT and Abx pikas. (B) NMDS plot based on Bray–Curtis distances representing the differences in the cecal microbial community structure among LP-FMT, LPY-FMT and Abx pikas. (C) Alpha diversity (Simpson index) of bacterial communities. Statistical significance was determined by one-way ANOVA with Tukey’s post-hoc test, where asterisks indicate a significant difference (p < 0.05) for the comparison among HP-FMT, MP-FMT, LP-FMT, and Abx groups; for the comparison among LP-FMT, LPY-FMT, and Abx groups, asterisks denote significance at three levels (*p < 0.05, **p < 0.01, ***p < 0.001). The numerical data used to generate the graphs in this figure are available in S13 Data. (TIF) [file pbio.3003436.s006.tif]

Figure 1

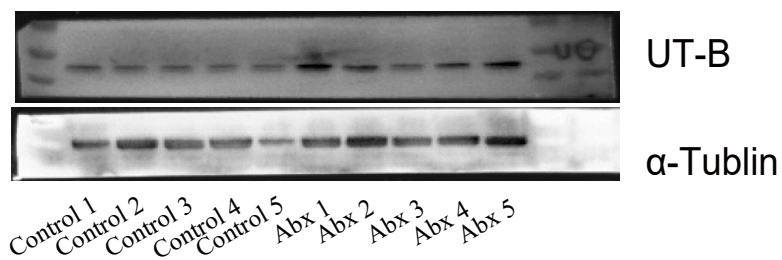

Figure 2

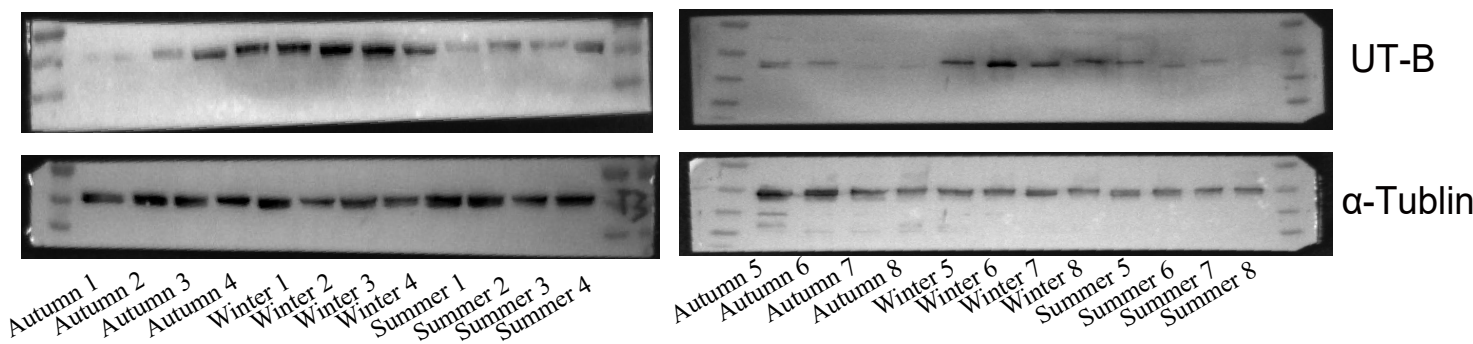

Figure 3 & 5

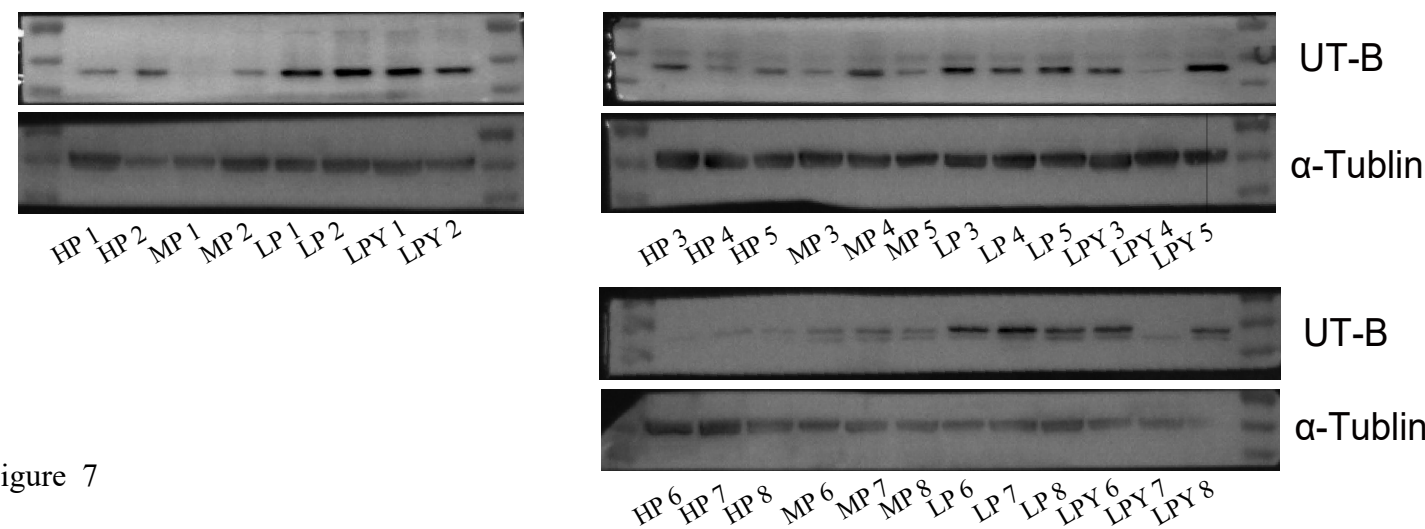

Figure 7

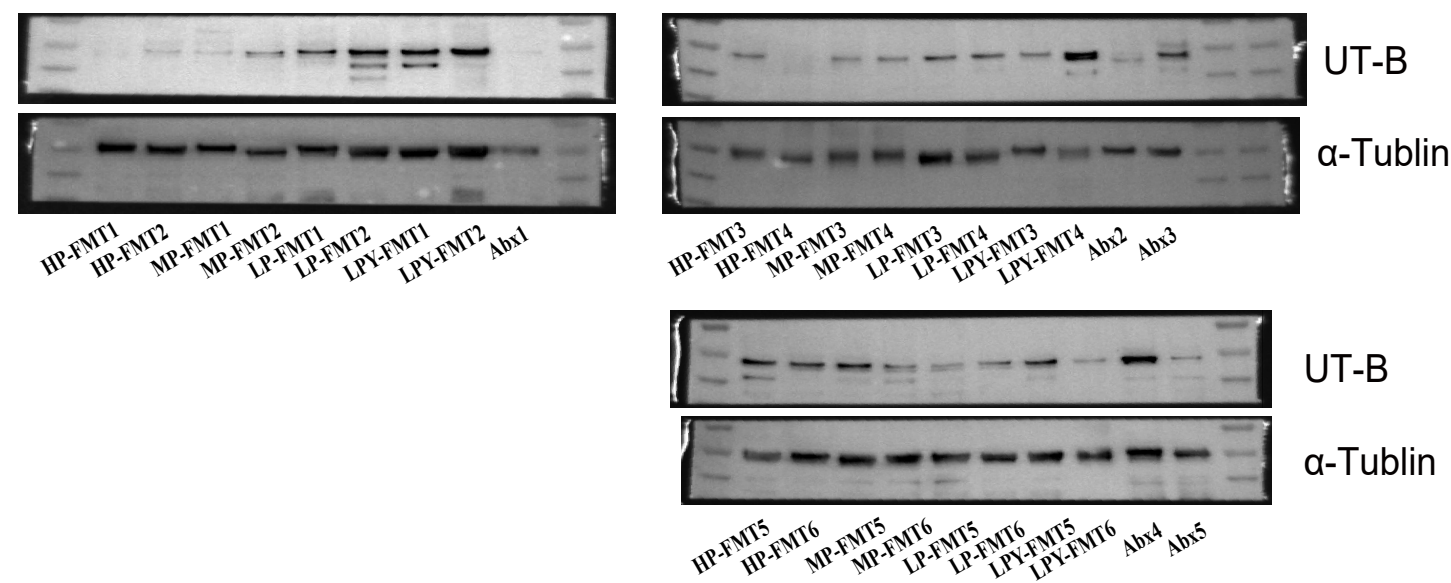

Supplement: S1 Raw Images — (PDF) [file pbio.3003436.s030.pdf]
