# Supplementary material for: Increased urea nitrogen salvaging by a remodeled gut microbiota helps nonhibernating pikas maintain protein homeostasis during winter
Source: PLoS Biol. 2025 Oct 16;23(10):e3003436. doi: 10.1371/journal.pbio.3003436 (PMC12530534; doi:10.1371/journal.pbio.3003436)
Supplement: S1 Table — (DOCX) [file pbio.3003436.s007.docx]

**S1 Table.** Protein content of the various diets

| Component | 6% CP | 12% CP | 18% CP |
| --- | --- | --- | --- |
| Corn | 14.0 | 14.0 | 14.0 |
| Subflour | 1.0 | 39.1 | 39.1 |
| Alfalfa meal | 0.0 | 25.0 | 35.0 |
| Grass meal | 46.0 | 1.0 | 4.0 |
| Microcrystalline cellulose | 16.7 | 14.6 | 0.0 |
| Casein | 0.0 | 0.0 | 3.0 |
| Soybean oil | 19.0 | 3.0 | 0.0 |
| Soybean meal | 0.0 | 0.0 | 0.6 |
| Calcium hydrogen phosphate | 2.0 | 2.0 | 2.0 |
| Yeast powder | 0.0 | 0.0 | 1.0 |
| 1% premix | 1.3 | 1.3 | 1.3 |
| Total | 100 | 100 | 100 |
| Crude protein, % | 6.0 | 12.0 | 18.0 |
| Crude fat, % | 20.1 | 5.6 | 3.0 |
| Carbohydrates, % | 10.1 | 36.0 | 36.1 |
| Gross energy, kcal/g | 2.4 | 2.4 | 2.4 |

CP: crude protein.
