# Supplementary material for: Increased urea nitrogen salvaging by a remodeled gut microbiota helps nonhibernating pikas maintain protein homeostasis during winter
Source: PLoS Biol. 2025 Oct 16;23(10):e3003436. doi: 10.1371/journal.pbio.3003436 (PMC12530534; doi:10.1371/journal.pbio.3003436)
Supplement: S2 Table — (DOCX) [file pbio.3003436.s008.docx]

**S2 Table.** Average relative abundance (%) of the dominant gut microbial phyla in pikas from the low-protein (LP) and supplementation of the diet with yak fecal bacteria (LPY) groups.

| **Phylum level** | **Average relative abundance (%)** | | ***p*-value** |
| --- | --- | --- | --- |
|  | **LP group** | **LPY group** |  |
| Bacillota | 73.20 | 67.43 | 0.122 |
| Bacteroidota | 16.10 | 21.32 | 0.034 |
| Actinomycetota | 3.86 | 3.72 | 0.696 |
| Pseudomonadota | 2.45 | 2.58 | 0.237 |
| Thermodesulfobacteriota | 2.00 | 2.24 | 0.083 |

Data are presented as mean ± SEM (n = 8 per group). The table shows the top 5 most abundant phyla. Statistical significance between the two groups was determined by the non-parametric Wilcox tests, significance was set at *p* < 0.05.
