# Supplementary material for: Increased urea nitrogen salvaging by a remodeled gut microbiota helps nonhibernating pikas maintain protein homeostasis during winter
Source: PLoS Biol. 2025 Oct 16;23(10):e3003436. doi: 10.1371/journal.pbio.3003436 (PMC12530534; doi:10.1371/journal.pbio.3003436)
Supplement: S3 Table — (DOCX) [file pbio.3003436.s009.docx]

**S3 Table.** Average relative abundance (%) of the dominant gut microbial family in pikas from the low-protein (LP) and supplementation of the diet with yak fecal bacteria (LPY) groups.

| **Phylum level** | **Average relative abundance (%)** | | ***p*-value** |
| --- | --- | --- | --- |
|  | **LP group** | **LPY group** |  |
| Oscillospiraceae | 43.44 | 42.82 | 0.481 |
| Lachnospiraceae | 18.95 | 14.65 | 0.093 |
| Prevotellaceae | 5.47 | 7.38 | 0.046 |
| Rikenellaceae | 2.78 | 3.99 | 0.036 |
| Bacteroidaceae | 2.94 | 3.75 | 0.093 |
| Clostridiaceae | 2.68 | 2.37 | 0.277 |
| Muribaculaceae | 2.03 | 2.69 | 0.093 |
| Paenibacillaceae | 1.75 | 1.59 | 0.139 |
| Desulfovibrionaceae | 1.43 | 1.68 | 0.139 |
| Aristaeellaceae | 0.99 | 0.93 | 0.815 |

Data are presented as mean ± SEM (n = 8 per group). The table shows the top 10 most abundant family. Statistical significance between the two groups was determined by the non-parametric Wilcox tests, significance was set at *p* < 0.05.
