# Supplementary material for: Increased urea nitrogen salvaging by a remodeled gut microbiota helps nonhibernating pikas maintain protein homeostasis during winter
Source: PLoS Biol. 2025 Oct 16;23(10):e3003436. doi: 10.1371/journal.pbio.3003436 (PMC12530534; doi:10.1371/journal.pbio.3003436)
Supplement: S4 Table — (DOCX) [file pbio.3003436.s010.docx]

**S4 Table.** Average relative abundance (%) of the dominant gut microbial genus in pikas from the low-protein (LP) and supplementation of the diet with yak fecal bacteria (LPY) groups.

| **Phylum level** | **Average relative abundance (%)** | | ***p*-value** |
| --- | --- | --- | --- |
|  | **LP group** | **LPY group** |  |
| *Flavonifractor* | 6.71 | 7.20 | 0.167 |
| *Dysosmobacter* | 6.46 | 6.03 | 0.481 |
| *Prevotella* | 3.64 | 4.93 | 0.059 |
| *Lawsonibacter* | 5.56 | 5.90 | 0.606 |
| *Flintibacter* | 3.92 | 4.06 | 0.815 |
| *Vescimonas* | 3.79 | 3.35 | 0.046 |
| *Faecalibacterium* | 3.35 | 3.21 | 0.481 |
| *Bacteroides* | 2.42 | 3.11 | 0.139 |
| *Alistipes* | 2.55 | 3.66 | 0.027 |

Data are presented as mean ± SEM (n = 8 per group). The table shows the top 10 most abundant genus. Statistical significance between the two groups was determined by the non-parametric Wilcox tests, significance was set at *p* < 0.05.
