# Supplementary material for: Increased urea nitrogen salvaging by a remodeled gut microbiota helps nonhibernating pikas maintain protein homeostasis during winter
Source: PLoS Biol. 2025 Oct 16;23(10):e3003436. doi: 10.1371/journal.pbio.3003436 (PMC12530534; doi:10.1371/journal.pbio.3003436)
Supplement: S5 Table — (DOCX) [file pbio.3003436.s011.docx]

**S5 Table**. Primer sequences used for quantitative PCR

| Gene |  | Primer sequence | Size |
| --- | --- | --- | --- |
| *Gapdh* | Forward | GGTCGGAGTGAACGGATTTGG | 214 |
|  | Reverse | GATGGTGATGGCTTTCCCGT |  |
| *Utb* | Forward | CACCTAATGGCTAGGGTTGGAT | 260 |
|  | Reverse | TCTTGGAAGTTGCATGGAGCA |  |
| *Asl* | Forward | CTCCACATTGCAGATTCACCG | 253 |
|  | Reverse | CACACTGTTCCCGTAGTCCC |  |
| *Otc* | Forward | GTGCGACATTTTGGGTGTGG | 262 |
|  | Reverse | AAGGGTGACCTCCCAGAAGT |  |
| *Ass1* | Forward | TCGGGGCCAAAAAGGTATTCA | 196 |
|  | Reverse | GTGGCACCATGAGACACGTA |  |
| *Cps1* | Forward | TGCATCCCGAGTTGAGGTTT | 256 |
|  | Reverse | GGGTCGTTCAGCCTTGATGA |  |
| *Snat1* | Forward | TGCTCCAAGGAGACAGGTTG | 291 |
|  | Reverse | GGTGTAGCCCAGGTACCCTA |  |
| *Snat3* | Forward | AGGTGGATCCATTCGACGTG | 248 |
|  | Reverse | AGCAGACGTAGCACCAATGA |  |
| *Lat2* | Forward | GCTGCCATCTGCTTATTGCT | 211 |
|  | Reverse | CGATGAGGCCAATGTCAGGT |  |
| *Lat1* | Forward | CGTTAGACTGGGCCAAGGAG | 176 |
|  | Reverse | ACAGGGGCAGATTCCTCTCA |  |
| *Eaat3* | Forward | GTGGGTGAGATCGACAGGAC | 128 |
|  | Reverse | CGCGAGGTTTTGTACTGCTG |  |
