# Supplementary material for: Increased urea nitrogen salvaging by a remodeled gut microbiota helps nonhibernating pikas maintain protein homeostasis during winter
Source: PLoS Biol. 2025 Oct 16;23(10):e3003436. doi: 10.1371/journal.pbio.3003436 (PMC12530534; doi:10.1371/journal.pbio.3003436)
Supplement: Attachment — Submitted filename: Response to Reviewers to PB_R1.docx [file pbio.3003436.s031.docx]

Dear Reviewers,

We are grateful to the Reviewers for their constructive comments and suggestions on our manuscript. We have carefully addressed all the points raised and believe the manuscript has been significantly strengthened as a result.

Our detailed point-by-point responses are provided below. We have highlighted the changes in the revised manuscript for your convenience.

**Point-by-point responses:**

Reviewer #1: The authors investigated how winter protein restriction affects hepatic urea nitrogen recycling in plateau pikas. Their findings demonstrate that gut microbiota-mediated urea nitrogen salvaging is enhanced under low-protein diets. Mechanistically, microbial urease hydrolyzes urea into CO_2_ and NH_3_, with the absorbed NH_3_ subsequently converted into amino acids for potential host uptake via amino acid transporters. While the overall logic is sound and the data generally support the conclusions, several aspects of the manuscript could be improved:

Major Points:

1. Figure 4 identifies differentially abundant microbial taxa between LP and HP groups using metagenomic analysis. Fecal microbiota transplantation (FMT) experiments with key microbial taxa are recommended to validate these metagenomic findings. Similarly, in Figure 6, FMT experiments should be conducted to confirm the role of differentially abundant microbes between LP and LPY groups.

**Response:** We sincerely thank the reviewer for this insightful and constructive feedback. We agree with your suggestion and recognize that using key taxa for functional validation is the ultimate goal for elucidating specific mechanisms. In fact, to functionally validate our metagenomic findings presented in Figures 4 and 6, we first performed the whole-community fecal microbiota transplantation (FMT) experiment, with the results shown in Figure 7. We observed that recipients of microbiota from the LP group exhibited enhanced urea-nitrogen salvaging compared to those receiving HP microbiota, and this effect was further enhanced in recipients of microbiota from the LPY group when compared to the LP group. These results indicate that the gut microbiota, as a whole, is involved in this important host adaptation to a LP environment. Since the differentially abundant taxa identified in Figures 4 and 6 are integral members of this community, these findings provide functional support for their role in the observed functional shifts. The experiment links the changes in the microbiota to the changes in host physiology, which supports the main conclusion of our paper.

Of course, we also agree that elucidating the deeper mechanisms requires moving to the key microbial strain level. The isolation and cultivation of specific functional strains from a unique host like the plateau pika is a significant and complex undertaking in its own right. We have, in fact, already initiated preliminary work on these key taxa and have designated this as a core component of our follow-up research plan. We have acknowledged this in the Discussion section of our manuscript, highlighting that validation with specific strains represents an important direction for future studies (**Line 362-366**). We hope that the evidence from our whole-community transplantation, combined with our clear plan for future research, adequately addresses your concerns. Thank you once again for your valuable feedback.

2.The microbial composition of the LPY group is suggested to explicitly describe. Additionally, the diversity of nitrogen-recycling microorganisms in this group should be characterized.

**Response:** We thank the reviewer for these valuable suggestions. We have revised the manuscript to provide a more explicit description of the LPY group's microbial composition and to characterize the diversity of its nitrogen-recycling microorganisms.

In the revised Results section (**Lines 196-207; Table S2-S4**), we have now added a detailed taxonomic and functional characterization.

**Microbial Composition:** We now explicitly describe the key taxonomic shifts in the LPY group, detailing the significant enrichment of the phylum Bacteroidota, driven by families such as Rikenellaceae, and specifically the genus *Alistipes*.

**Diversity of Nitrogen-Recycling Microorganisms:** To characterize the functional diversity, we have incorporated our metagenomic data. We now specify the key urease-encoding MAGs (e.g., *Alistipes* sp. and *CAG-485* sp. bins) that were more abundant in the LPY group. Crucially, we have also included new results from an Upset plot analysis, which quantifies the diversity at the gene level. This analysis demonstrates that the LPY group possesses a more diverse repertoire of urease homologs (28 unique variants) compared to the LP group (21 unique variants).

3.How the nitrogen-recycling microorganisms are increased under LP or winter conditions?

**Response:** We thank the reviewer for this excellent question. The increase of nitrogen-recycling microbiota under LP or winter conditions is due to two main reasons: the environment favoring them and the pika’s behavior of acquiring them. We have clarified this in the Discussion section (**Lines 298-301 and Line 338-344**).

1. The LP environment gives them an advantage:

A LP diet means there is very little nitrogen available in the gut. Most bacteria need nitrogen to grow, so this environment makes it hard for them to survive. However, certain bacteria have special enzymes, like urease, which allow them to use the pika's own waste product (urea) as a nitrogen source. This gives them a significant competitive advantage. In this environment, they can grow and multiply more successfully than other bacteria that lack this ability.

2. Pikas actively eat bacteria-rich feces:

Pikas don't just wait for their own gut bacteria to adapt. Our study shows that they actively eat yak feces. Yak feces contain a large number of bacteria that are very efficient at recycling nitrogen, such as *Alistipes*. By eating yak feces, pikas directly introduce these highly effective microbes into their gut.

The overall process is: The low-protein diet creates an environment where nitrogen-recycling bacteria are more likely to succeed. Pikas eat yak feces, which provides a direct source of these beneficial bacteria. These newly introduced bacteria are better at surviving in the pika's gut than the pika's original bacteria. They successfully establish themselves, grow in number, and become a dominant part of the gut community.

In short, the increase in these microorganisms is a combination of favorable environmental conditions and an active behavioral strategy by the pikas to acquire the right microbes for that environment.

4.Additional details regarding experimental conditions, including ambient temperature, environmental sterility levels, and antibiotic treatments administered to the animals, are suggested to describe.

**Response:** We sincerely thank the reviewer for this constructive suggestion, which is crucial for ensuring the transparency and reproducibility of our study. Following the suggestion, we have provided a more detailed description of the experimental conditions in the Materials and Methods section of the manuscript. This includes additional details on the ambient temperature, environmental sterility levels, and the antibiotic treatments administered to the animals, and also expand descriptions of key analytical measurements, such as the quantification of SCFAs and the ^13^C and ^15^N isotope tracing and analysis.

Specifically, we provide the following details as follows:

**Ambient Temperature and Conditions:** All animals were housed under strictly controlled environmental conditions. Specifically, the room was maintained under controlled conditions, including a room temperature of 12-15℃ (reflecting the natural temperature of the experimental environment) and a 12-h light/dark cycle **(Line 391-394)**.

**Environmental Sterility Levels:** Upon arrival, they were immediately housed individually in standard cages (50 cm × 35 cm × 20 cm) and were allowed a 3-weeks acclimation period to ensure physiological stabilization. This entire period was conducted within a dedicated animal room that was thoroughly disinfected prior to the experiment **(Line 388-391)**. To minimize cross-contamination, all cages, food, and water were sterilized, and strict hygiene protocols were enforced with restricted personnel access. All animals were monitored daily and remained healthy, showing no signs of infection or distress throughout the experimental period **(Line 394-395)**.

**Antibiotic Treatments:** We have now elaborated on the antibiotic treatment in the manuscript. The antibiotic cocktail used for microbiota depletion consisted of streptomycin (50 μg/mL), neomycin (100 μg/mL), and penicillin (100 U/mL) (all from Sigma, Germany), dissolved in sterile saline. A precise volume of 400 μL of this mixture was administered to each animal via oral gavage. For clarity, the specific administration details, including the duration and route (400 μL via oral gavage), are described within the methods for each relevant experiment (**Experiment 1: Line 403-406; and Experiment 5: Line 452-462**).

**Other key analytical measurements:** the “Breath analysis” (**Line 467-478**), “Analysis of ^15^N incorporation into protein” (**Line 481-510**) and “Quantification of SCFAs” (**Line 581-597**).

We believe these revisions ensure our experimental methods can be clearly understood and replicated. Thank you again for your insightful feedback.

**Minor Points:**

5. While plasma and cecal urea concentrations were measured, the manuscript does not report urine urea levels. Including these data would provide a more comprehensive assessment of nitrogen excretion and recycling dynamics.

**Response:** We would like to thank the reviewer for this valuable suggestion. We agree that incorporating the analysis of urine urea would indeed offer a more comprehensive assessment of the nitrogen balance, and we appreciate you highlighting this comprehensive approach.

In the current study, our primary focus was to specifically elucidate the host-microbiota axis of nitrogen recycling, for which plasma and cecal urea levels are the most direct and crucial indicators. To avoid potential alterations in metabolic parameters caused by the stress associated with urine collection, this step was omitted from this study. However, we do recognize the contribution of urine urea to host nitrogen balance and plan to incorporate this measurement in subsequent studies for a more comprehensive assessment.

We recognize the value of your point and have now explicitly acknowledged this as a limitation in our Discussion section. We have added a new section to our Discussion **(Line 272-273)**. We agree that while our study focused on plasma and cecal urea, including urine urea analysis in future studies would indeed provide a more complete picture of whole-body nitrogen balance. We thank you for suggesting this important avenue for future research.

6. To assess seasonal variations in urea nitrogen recycling, pikas were captured across different seasons. However, it is unclear whether pikas in different experimental groups were age- and weight-matched. This information is critical for interpreting group differences.

**Response:** We thank the reviewer for raising this critical point. The concern regarding the matching of age and weight across different experimental groups is indeed crucial for the accurate interpretation of our findings. Our approach to controlling these variables was based on previous research from our laboratory. Our prior studies have established that the body weight of plateau pikas remains relatively stable across different seasons (Wang and Wang, 1990; Wang et al., 2006; Speakman et al., 2021). Furthermore, in adult plateau pikas, body weight serves as a reliable indicator of age (Nie et al., 2022).

Accordingly, to minimize the influence of age and body weight differences, we applied a strict selection rule: only pikas with a body weight between 130-150 g were included in our experiments. This narrow weight range helped ensure that any observed physiological changes were due to seasonal effects, rather than differences in age or initial weight among the groups. We have now added this crucial selection criterion to the Materials and Methods section of the manuscript to make our experimental design clearer **(Line 418-421).** Thank you again for your constructive feedback.

**Relevant references:**

Wang DH, Wang ZW. Strategies for survival of small mammals in a cold alpine environment II. Seasonal changes in the capacity of nonshivering thermogenesis in *Ochotona curzoniae* and *Microtus oeconomus*. Acta Theriologica Sinica. 1990;10(1):40-53.

Wang JM, Zhang YM, Wang DH. Seasonal thermogenesis and body mass regulation in plateau pikas (*Ochotona curzoniae*). Oecologia. 2006;149(3):373-382.

Speakman JR, Chi QS, Oldakowski L, Fu HB, Fletcher QE, Hambly C, et al. Surviving winter on the Qinghai-Tibetan Plateau: Pikas suppress energy demands and exploit yak feces to survive winter. Proceedings of the National Academy of Sciences. 2021;118(30): e2100707118.

Nie H, Liu J, Chen B. Life‐history traits and fitness of plateau pika (*Ochotona curzoniae*) in alpine meadow ecosystem. Ecology and Evolution. 2022;12(1):e8548.

7.In the Results section, the diet compositions are incorrectly stated as: "HP (high protein), containing 18% crude protein (CP); MP (medium protein), 12% CP; and LP, 18% CP." This appears to be a typo. Based on Figure 3, the LP group should contain 6% CP.

**Response:** We appreciate your careful reading that caught this error. The text has now been revised to accurately state that the crude protein content for the LP group is 6%, consistent with our experimental design and other figures.

8.Western blot (WB) group labels are missing in Figures 3m, 5I and 7I. These should be added for clarity.

**Response:** Thank you for pointing this out. We have now added the missing group labels to Figures 3m, 5I, and 7I in the revised manuscript for better clarity. We appreciate your careful review.

Reviewer #2: This manuscript presents a case study demonstrating enhanced gut microbiota-mediated urea nitrogen salvaging in response to a low-protein diet during winter in a non-hibernating small mammal. The plateau pika (*Ochotona curzoniae*), a small lagomorph inhabiting the alpine meadows of the Qinghai-Tibetan Plateau, survives the long, cold winter in the face of forage with low protein content. The authors hypothesized that pikas maintain protein homeostasis by enhancing urea nitrogen recycling, particularly during winter when forage protein content is low. Furthermore, winter coprophagy of sympatric yak feces provides plateau pikas with essential nutrients and energy while concurrently altering their gut microbiota composition. Consequently, the authors proposed that this coprophagous behavior increases urea nitrogen utilization, facilitating protein homeostasis maintenance throughout winter. Through integrated application of isotope tracing, metagenomics, and fecal microbiota transplantation, the authors validated their hypotheses. Overall, this study exemplifies a clearly defined scientific question, robust experimental design, and integrated multiple methodologies, resulting in a comprehensive investigation. However, I do have some questions and comments below.

Line 80-83: Is this interspecific coprophagy behavior limited to winter? I suggest you could appropriately introduce a bit more about this behavior. Additional background information on this phenomenon would strengthen the context.

**Response:** Thank you for this excellent suggestion. We agree that providing additional background information on the interspecific coprophagy of plateau pikas significantly strengthens the biological context of our study. Based on our observations and the available literature, this behavior does indeed appear to be a seasonal strategy, primarily observed during the winter when food resources are scarce. Following your advice, we have expanded on this phenomenon in the Introduction section **(Lines 80-94)** and Discussion section (**Lines 352-355**) of our revised manuscript. The new text now includes more details on the ecological context, the evidence supporting this behavior, and its potential multifaceted benefits (e.g., nutritional gain), which provides a much stronger foundation for our central hypothesis.

Line 86-90: I recommend refining the hypothesis to sharpen the research focus and avoid a contrived nature. You presented three hypotheses. However, the first two appear to lack sufficient novelty based on current literature for all I know. And the interspecific coprophagy phenomenon represents your most distinctive finding. It may be more attractive for most researchers within the field of zoology, animal behavior, and gut metagenomics, to uncover the role of interspecific corprophagy. You could further develop hypotheses integrating environmental stress (e.g., nitrogen balance), animal behavior (e.g., foraging and interspecific coprophagy), and gut microbiota into a comprehensive theoretical framework.

**Response:** We sincerely thank the reviewer for this critical and insightful comment. We agree with your assessment and appreciate your guidance on how to build a more compelling and integrated theoretical framework. As you mentioned, our initial presentation of three separate hypotheses failed to highlight the central importance and novelty of the interspecific coprophagy phenomenon. Following your invaluable suggestion, we have completely restructured our hypotheses in the Introduction (**Lines 95-103**). We now propose a single, central hypothesis that integrates the key elements you mentioned (environmental stress, animal behavior, and gut microbiota) and three specific predictions.

Our new hypothesis is now stated as: “This central hypothesis leads to three specific predictions: (1) the native gut microbiota of pika possesses an inherent capacity for urea-nitrogen recycling, and that this function is significantly upregulated in response to a low-protein (LP) diet; (2) pikas use interspecific coprophagy (experimentally simulated by supplementing an LP diet with yak fecal microbiota, i.e., the LPY diet) as a behavioral strategy to further augment this inherent capacity, by acquiring potent ureolytic microbes from yak feces; (3) this microbial enhancement translates into a physiological benefit, namely the improved maintenance of protein homeostasis.” Thank you once again for your insightful guidance, which has been instrumental in improving our manuscript.

Line 114: The protein content of LP group is 6%.

**Response:** Thank you very much for the correction. We have corrected the protein content of the LP group to 6% in the manuscript.

Line 201-203: The α-diversity, as determined by species richness, was lowest in LP-FMT pikas, whereas the Simpson index did not differ (p>0.05) among the FMT-HP, FMT-MP and FMT-LP groups. However, the α-diversity, based on species richness, was higher in the LP than HP group. How do you interpret this phenomenon? Could it be related to the effectiveness of FMT? How do you evaluate the consequence of FMT and its impact on the conclusion?

**Response:**

(1) How do you interpret this phenomenon?

**Why was richness higher in the LP group than the HP group (without FMT)?** A LP diet represents a state of nutrient limitation. In such conditions, the host's gut may need to maintain a more diverse microbial community to acquire nitrogen and efficiently break down complex plant fibers. This functional redundancy and metabolic diversity are reflected as higher species richness. On the other hand, a HP diet offers plenty of easily accessible food. This can allow a few very competitive species that excel at breaking down protein to take over, reducing the variety of microbes through competition.

**Why did LP-FMT pikas have the lowest richness?** Gut bacteria from yaks, particularly a type called *Alistipes*, are highly efficient at breaking down urea. This ability is essential for them to survive on a LP diet. When these highly effective bacteria are transplanted into pikas, their efficiency makes them extremely competitive. They successfully colonize and proliferate, outcompeting many of the resident, less-efficient microbes.

(2) Could it be related to the effectiveness of FMT?

The objective of this FMT was not simply to increase microbial diversity. It was to transfer a specific ecological function: the ability to recycle urea nitrogen. In our study, we found ^15^N was successfully incorporated into microbial and host proteins, confirming the function was enhanced. The result that richness decreased in the LP-FMT group is evidence that the transplanted microbes were not merely transient, but demonstrated successful colonization. They reshaped the community structure of gut microbiota. If the FMT had been ineffective, the transplanted microbes would have failed to establish, and the richness would have remained high and similar to the LP control group. The fact that the Simpson index did not differ significantly among the FMT groups is also telling. The Simpson index accounts for evenness of gut microbiome. This suggests that while the number of species dropped (richness), the community didn't collapse into being dominated by a single species. Rather, it was remodeled into a stable and functionally coherent community with a different composition but similar evenness.

(3) How do you evaluate the consequence of FMT and its impact on the conclusion?

Our interpretation is that the transplanted yak microbes, being highly efficient at recycling nitrogen, successfully colonized the pika's gut. They thrived in the low-protein environment and outcompeted many of the less-efficient resident microbes, which caused the total number of species to decrease. The stable Simpson index confirms this was a healthy change: the new community remained well-balanced.

This directly impacts our conclusion. It proves that the FMT successfully transferred a critical function (urea-nitrogen recycling). Therefore, pikas eating yak fecal is more than just simple feeding—it's a remarkably strategy to acquire beneficial microbes. This finding provides the evidence linking this behavior to the pikas' enhanced survival and the positive population correlation observed in the wild.

Line 373-377: How many individuals do these ten fecal samples originate from?

**Response:** Thank you for requesting this clarification. The ten fecal samples were collected from ten different individual adult yaks. To ensure sample independence, we followed a herd of yaks and collected fresh fecal samples immediately after excretion. We have now added this specific detail to the Materials and Methods section of the revised manuscript to ensure clarity **(Line 440-441**).

Line 502-508: The methodology in this section could be more detailed. It is necessary to provide the information on standard compounds, and mass spectral target peak in the supporting attachment. Furthermore, given that only six short-chain fatty acids (SCFAs) were analyzed, the use of "metabolomics" in the abstract appears inappropriate. Metabolomic studies typically characterize global metabolic profiles through the quantification of numerous compounds, extending well beyond SCFAs alone.

**Response:** We sincerely thank the reviewer for these valuable suggestions. We agree that more details are needed for methodological clarity and that the term "metabolomics" should be used more precisely. We have addressed these points as follows:

**1. In the main text:** We have expanded the Methodology section **(Line 581-597)** to include detailed information about the six standard compounds, such as their names, purity, and suppliers.

**2. In the** **Supporting Information:** As requested, we have created a new table (Supplementary Table S1) which provides the specific mass spectral target peaks for each SCFA, including precursor/product ion pairs (m/z) and retention times **(Supplementary dataset 6)**.

**3. Regarding the use of the term "metabolomics":** We agree that our targeted analysis of six SCFAs does not constitute a full metabolomics study. We have carefully revised the entire manuscript and replaced the term "metabolomics" with more appropriate phrases, such as “SCFA quantification”. This change has been implemented in the abstract, result, and all other relevant sections.

Figures and tables: There are some minor spelling mistakes inside the figures (e.g., Fig. 4f; Fig. 6; Fig.7, etc). What’s the meaning of the group information "CK-FMT" in Fig. 7o? Is it referring to Abx? In addition, consistency in statistical annotations is essential throughout the manuscript. It is not recommended that you used different marking of statistical significance in one paper, especially in a specific figure or subfigure, to ensure clarity and avoid confusion. It is clear enough to use asterisk system (*p < 0.05; **p < 0.01; ***p < 0.001) to display the degree of deviation. Overall, both the figures and the tables should be self-explanatory. All figures (including those in the supplementary information) should have titles and a brief legend explaining their content. Specifically, current supplementary figures lack sufficient legend detail and require expansion for clarity.

**Response:** Thank you for your thorough and highly valuable feedback on our figures and tables. We completely agree that clear, consistent, and self-explanatory figures are fundamental to a high-quality manuscript. We have performed a comprehensive revision of all figures and tables based on your excellent suggestions.

Our specific revisions are as follows:

**Spelling Mistakes:** We apologize for the oversight. We have carefully proofread all figures (including the examples you noted in Fig. 4f, 6, and 7) and have corrected all spelling errors.

**"CK-FMT" Group Label in Fig. 7o:** Your question is spot on. "CK-FMT" was indeed our abbreviation for the control group, which refers to the mice that were treated with antibiotics followed by a sham transplantation with sterile saline. We acknowledge that this abbreviation was not clear and could cause confusion. To eliminate any ambiguity, we have replaced "CK-FMT" with "Abx".

**Consistency in Statistical Annotations:** We have now standardized the statistical annotations throughout the entire manuscript (including all main and supplementary figures). We now exclusively use the asterisk system (*p < 0.05; **p < 0.01; ***p < 0.001) that you suggested to indicate levels of statistical significance, ensuring consistency and clarity.

**Self-Explanatory Figures and Legends:** We recognize the importance of making all figures and tables self-explanatory, particularly those in the supplementary information. We have conducted a thorough review and revision. Every figure and table now have a clear title and a detailed, self-contained legend. Specifically for the supplementary figures, we have significantly expanded the legends to include essential details such as the number of biological replicates (n), the statistical tests performed, and the definition of error bars (e.g., mean ± SEM).

We wish our careful revision can match the requirements for publication in *PLOS Biology*. If still have some questions, please let us know soon.

**Sincerely yours,**

Fuyu Shi (202290900079@sdu.edu.cn)

Yanming Zhang (zhangym@nwipb.cas.cn)

Dehua Wang (dehuawang@sdu.edu.cn)
