# Supplementary material for: Increased urea nitrogen salvaging by a remodeled gut microbiota helps nonhibernating pikas maintain protein homeostasis during winter
Source: PLoS Biol. 2025 Oct 16;23(10):e3003436. doi: 10.1371/journal.pbio.3003436 (PMC12530534; doi:10.1371/journal.pbio.3003436)
Supplement: Attachment — Submitted filename: Response to Editor.docx [file pbio.3003436.s032.docx]

**Dear Editor:**

We are very grateful to the editors for their positive assessment of our manuscript and for providing this clear list of final requests. We have addressed all points as detailed below. The editor’s original comments are shown in bold, followed by our response.

**Response to Editorial Requests:**

**1) TITLE: After discussing the title of your paper within the team, we are wondering if it could be improved by including discussion of the relevance of the increased microbiota mediated nitrogen salvaging. We also think it would be good to indicate that this is in non-hibernating animals. Therefore, if you agree this is supported and appropriate, we suggest you change the title to something like:**

**"Increased urea nitrogen salvaging by a remodeled gut microbiota helps non-hibernating pikas survive their low-protein winter diet"**

**Response:** Thank you for this excellent and insightful suggestion. We completely agree that the proposed title significantly improves the manuscript.

The suggested title, "Increased urea nitrogen salvaging by a remodeled gut microbiota helps non-hibernating pikas survive their low-protein winter diet," is more impactful and informative. It succinctly encapsulates not only our core findings—the mechanism of nitrogen salvaging and the role of the gut microbiota—but also crucially highlights the broader ecological significance of this adaptation for the survival of a non-hibernating animal in a harsh environment. We believe this title will better attract the interest of a broad readership.

We have changed the title in our revised manuscript accordingly. Thank you once again for your constructive feedback.

**2) FINANCIAL DISCLOSURES: Please update your financial disclosures statement, in our editorial manager system, to include the funder websites and a statement indicating whether the sponsors or funders play any role in the study design, data collection and analysis, decision to publish, or preparation of the manuscript.**

**Response:** Thank you for the reminder. We have now updated our financial disclosure statement in the editorial manager system as requested.

The updated statement now includes the websites for all our funders. Furthermore, we have added the required statement confirming that the sponsors or funders had no role in the study design, data collection and analysis, the decision to publish, or the preparation of the manuscript.

**3) ETHICS STATEMENT:**

**-- Please include the specific national or international regulations/guidelines to which your animal care and use protocol adhered. Please note that institutional or accreditation organization guidelines (such as AAALAC) do not meet this requirement.**

**-- I see that you collected wild animals for your study. Please provide the relevant field license number and information on which body granted approval.**

**Response:** Thank you for the opportunity to provide further details on our animal care and ethical approvals. We have revised the manuscript to include the specific information you requested.

In the appropriate section of our Materials and Methods (Line 382-383), we have now explicitly stated the specific national guidelines to which our animal care and use protocol adhered.

Furthermore, we have added the field license number for the collection of wild animals and clearly identified the governing body that granted this approval (Line 383-384).

We trust this fully addresses the journal's requirements for ethical reporting.

**4) DATA: Please deposit your raw metagenomic sequencing data on a publicly available repository and update your data availability statement to reference that deposition. （已完成）**

**Response:** Thank you for your instructions. We have made our data publicly available and updated the manuscript accordingly.

We have deposited the raw metagenomic sequencing reads in the Sequence Read Archive (SRA) of the National Center for Biotechnology Information (NCBI). The data are available under the BioProject accession number PRJNA1190225. Direct access for review is available at: https://dataview.ncbi.nlm.nih.gov/object/PRJNA1190225?reviewer=4q92h87i30nqhcmmk7h6heo64s.

In addition, and to ensure full transparency and reproducibility, the Metagenome-Assembled Genomes (MAGs) and their functional annotation files generated from this data have been deposited in the Figshare repository under the DOI: 10.6084/m9.figshare.29646257.

We have updated the Data Availability Statement in the revised manuscript to include these accession numbers, the DOI, and the relevant links (Line 622-630).

**5) DATA: Additionally, you may be aware of the PLOS Data Policy, which requires that all data be made available without restriction: http://journals.plos.org/plosbiology/s/data-availability. For more information, please also see this editorial: http://dx.doi.org/10.1371/journal.pbio.1001797**

**Note that we do not require all raw data. Rather, we ask that all individual quantitative observations that underlie the data summarized in the figures and results of your paper be made available in one of the following forms:**

**a. Supplementary files (e.g., excel). Please ensure that all data files are uploaded as 'Supporting Information' and are invariably referred to (in the manuscript, figure legends, and the Description field when uploading your files) using the following format verbatim: S1 Data, S2 Data, etc. Multiple panels of a single or even several figures can be included as multiple sheets in one excel file that is saved using exactly the following convention: S1_Data.xlsx (using an underscore).**

**b. Deposition in a publicly available repository. Please also provide the accession code or a reviewer link so that we may view your data before publication.**

**---Regardless of the method selected, please ensure that you provide the individual numerical values that underlie the summary data displayed in the following figure panels as they are essential for readers to assess your analysis and to reproduce it:**

**Fig 1B-C,E-J; Fig 2B-E; Fig 3B-Q; Fig 4A-B; Fig 5B-P; Fig 6A-D; Fig 8B-O;**

**FIg S1A-J; Fig S2A,C; Fig S3; Fig S5A-K; FIg S5A-J; Fig S6A-C;**

**NOTE: the numerical data provided should include all replicates AND the way in which the plotted mean and errors were derived (it should not present only the mean/average values).**

**---Please also ensure that figure legends in your manuscript include information on where the underlying data can be found, and ensure your supplemental data file/s has a legend.**

**----Please ensure that your Data Statement in the submission system accurately describes where your data can be found.**

**(I see that you do have some data files already accompanying your manuscript, so apologies if some of these data are already provided. Please update these files to make it clear which figure panels they relate to).**

**Response:** Thank you for providing these detailed instructions regarding the PLOS Data Policy. We fully understand and support the importance of making the underlying data for our study publicly accessible. We apologize for any initial oversight and have now taken comprehensive steps to ensure full compliance.

In accordance with your instructions, we have chosen option (a) and have prepared supplementary data files containing the individual numerical values for all requested figures.

Our specific actions are as follows:

Provision of Underlying Data: We have compiled all individual quantitative observations for the specified figure panels (Fig 1B-C,E-J; Fig 2B-E; Fig 3B-Q; Fig 4A-B; Fig 5B-P; Fig 6A-D; Fig 8B-O; FIg S1A-J; Fig S2A,C; Fig S3; Fig S5A-K; Fig S6A-C) into supplementary Excel files.

Data Format and Naming: These files have been formatted and named strictly following the journal's convention (e.g., S1 Data.xlsx). To ensure clarity, we have organized the data into separate sheets within each Excel file, with each sheet clearly labeled to correspond to a specific figure panel. Crucially, these files contain the raw numerical data for all replicates, not just the summarized mean and error values.

Figure Legend Updates: We have meticulously revised the legends for every figure in the manuscript to include a sentence that explicitly directs the reader to the location of the underlying data.

Data Availability Statement Update: We have carefully reviewed and updated the Data Availability Statement in the submission system to accurately and clearly state that all relevant data are available within the paper and its Supporting Information files.

We have reviewed our existing data files and updated them as needed to ensure it is immediately obvious which data corresponds to which figure panel. We are confident that these actions fulfill all the requirements of the PLOS Data Policy and will allow readers to fully assess and reproduce our analyses.

Thank you again for your clear guidance.

**6) BLOT AND GEL REPORTING REQUIREMENTS: Please note that we require the original, uncropped and minimally adjusted images supporting all blot and gel results reported in an article's figures or Supporting Information files. We will require these files before a manuscript can be accepted so please prepare and upload them now. Please carefully read our guidelines for how to prepare and upload this data: https://journals.plos.org/plosbiology/s/figures#loc-blot-and-gel-reporting-requirements**

**Response:** Thank you for the reminder and for providing the link to the journal's blot and gel reporting requirements.

In accordance with your request, we have now prepared and uploaded the original, uncropped, and minimally adjusted images for all blot and gel results presented in our manuscript (S1 Raw Images).

We trust this fulfills the journal's requirements. Please let us know if any further information is required.

**7) CODE: Per journal policy, if you have generated any custom code during the course of this investigation, please make it available without restrictions. Please ensure that the code is sufficiently well documented and reusable, and that your Data Statement in the Editorial Manager submission system accurately describes where your code can be found.**

**Please note that we cannot accept sole deposition of code in GitHub, as this could be changed after publication. However, you can archive this version of your publicly available GitHub code to Zenodo. Once you do this, it will generate a DOI number, which you will need to provide in the Data Accessibility Statement (you are welcome to also provide the GitHub access information). See the process for doing this here: https://docs.github.com/en/repositories/archiving-a-github-repository/referencing-and-citing-content**

**Response:** Thank you for your clear instructions regarding the journal's code availability policy. We understand the importance of ensuring that any custom code is permanently and openly available for reproducibility.

Following your recommendation, we have archived the GitHub repository containing all custom code used for the analyses in this manuscript on Zenodo. The code is documented to facilitate reusability.

The archived code is now permanently available and can be accessed via the following DOI: https://doi.org/10.5281/zenodo.17120073

We have updated the Data Availability Statement in our manuscript and in the submission system to include this Zenodo DOI, as well as the link to the original GitHub repository for user convenience.

We trust this meets the journal's requirements for code sharing. Please let us know if any further action is required on our part.

**As you address these items, please take this last chance to review your reference list to ensure that it is complete and correct. If you have cited papers that have been retracted, please include the rationale for doing so in the manuscript text, or remove these references and replace them with relevant current references. Any changes to the reference list should be mentioned in the cover letter that accompanies your revised manuscript.**

**Response:** Thank you for the reminder and for providing this final opportunity to review our reference list.

We have conducted a thorough check of all citations for completeness and correctness. During this review, we confirmed that we have not cited any retracted papers. We did identify and correct some minor formatting issues to ensure full consistency with the journal's style guidelines.

As requested, we will note these minor formatting corrections in the cover letter that accompanies our revised manuscript. We are confident that the reference list is now complete, accurate, and correctly formatted.

**In addition to these revisions, you may need to complete some formatting changes, which you will receive in a follow up email. A member of our team will be in touch with a set of requests shortly. If you do not receive a separate email within a few days, please assume that checks have been completed, and no additional changes are required.**

**Response:** Thank you for the notification. We will keep an eye out for any further instructions on formatting

**To submit your revision, please go to https://www.editorialmanager.com/pbiology/ and log in as an Author. Click the link labelled 'Submissions Needing Revision' to find your submission record. Your revised submission must include the following:**

**- a cover letter that should detail your responses to any editorial requests, if applicable, and whether changes have been made to the reference list**

**- a Response to Reviewers file that provides a detailed response to the reviewers' comments (if applicable, if not applicable please do not delete your existing 'Response to Reviewers' file.)**

**- a track-changes file indicating any changes that you have made to the manuscript.**

**Response:** Thank you for providing these clear and detailed instructions for the submission process. We will follow them carefully and ensure our revised submission includes the cover letter, the Response to Reviewers file, and the track-changes manuscript, all uploaded through the Editorial Manager portal.

**NOTE: If Supporting Information files are included with your article, note that these are not copyedited and will be published as they are submitted. Please ensure that these files are legible and of high quality (at least 300 dpi) in an easily accessible file format. For this reason, please be aware that any references listed in an SI file will not be indexed. For more information, see our Supporting Information guidelines:**

**https://journals.plos.org/plosbiology/s/supporting-information**

**Response:** Thank you for this important reminder about the Supporting Information files.

We have carefully reviewed all of our Supporting Information files to ensure they are legible, formatted correctly, and meet the high-quality standard (at least 300 dpi) as required by the journal's guidelines.

***Published Peer Review History***

**Please note that you may have the opportunity to make the peer review history publicly available. The record will include editor decision letters (with reviews) and your responses to reviewer comments. If eligible, we will contact you to opt in or out. Please see here for more details:**

**https://plos.org/published-peer-review-history/**

**Response:** Thank you for this information. We understand the process for published peer review and will await further contact from you regarding this option.

***Press***

**Should you, your institution's press office or the journal office choose to press release your paper, please ensure you have opted out of Early Article Posting on the submission form. We ask that you notify us as soon as possible if you or your institution is planning to press release the article.**

**Response:** Thank you for the information regarding the journal's press release policy.

At this time, neither we nor our institution are planning to issue a press release for this article.

***Protocols deposition***

**To enhance the reproducibility of your results, we recommend that if applicable you deposit your laboratory protocols in protocols.io, where a protocol can be assigned its own identifier (DOI) such that it can be cited independently in the future. Additionally, PLOS ONE offers an option for publishing peer-reviewed Lab Protocol articles, which describe protocols hosted on protocols.io. Read more information on sharing protocols at https://plos.org/protocols?utm_medium=editorial-email&utm_source=authorletters&utm_campaign=protocols**

**Response:** Thank you for the recommendation regarding protocols.io and for the information on publishing Lab Protocols. We fully support the journal's commitment to enhancing reproducibility.

For the current submission, we believe that the detailed descriptions already provided in our Materials and Methods section are sufficiently comprehensive to allow other researchers to replicate our work. We will, however, certainly keep this valuable platform in mind for our future studies.
